# Ecologic Determinants of West Nile Virus Seroprevalence among Equids, Brazil

**DOI:** 10.3201/eid2709.204706

**Published:** 2021-09

**Authors:** Edmilson F. de Oliveira-Filho, Carlo Fischer, Beatrice Sarah Berneck, Ianei O. Carneiro, Arne Kühne, Angelica C. de Almeida Campos, Jorge R.L. Ribas, Eduardo Martins Netto, Carlos Roberto Franke, Sebastian Ulbert, Jan Felix Drexler

**Affiliations:** Charité-Universitätsmedizin Berlin, Berlin, Germany (E.F. de Oliveira-Filho, C. Fischer, A. Kühne, A.C. de Almeida Campos, J.F. Drexler);; Fraunhofer Institute for Cell Therapy and Immunology, Leipzig, Germany (B.S. Berneck, S. Ulbert);; Federal University of Bahia, Salvador, Brazil (I.O. Carneiro, E.M. Netto, C.R. Franke);; Bahia State Agricultural Defense Agency, Salvador (J.R.L. Ribas);; Sechenov University, Moscow, Russia (J.F. Drexler);; German Centre for Infection Research, Berlin (J.F. Drexler)

**Keywords:** West Nile virus, mosquitoborne disease, vector-borne infections, flaviviruses, equids, meningitis/encephalitis, mosquitoes, horses, viruses, zoonoses, Brazil

## Abstract

Among 713 equids sampled in northeastern Brazil during 2013–2018, West Nile virus seroprevalence was 4.5% (95% CI 3.1%–6.3%). Mathematical modeling substantiated higher seroprevalence adjacent to an avian migratory route and in areas characterized by forest loss, implying increased risk for zoonotic infections in disturbed areas.

West Nile virus (WNV) is a widely distributed arthropodborne flavivirus transmitted predominantly by *Culex* mosquitoes (*1*). Among infected persons, ≈20% show clinical signs, such as mild fever, rash, joint pain, headache, vomiting, and diarrhea (*1*,*2*); ≈0.7% have severe illness, such as encephalitis, meningitis, acute flaccid paralysis, respiratory failure, and even death (*1*). Beyond vectorborne transmission, transfusion-transmitted WNV infections have endangered blood safety (*3*). Equids are susceptible to WNV and develop severe disease (fatality rate <30%), are exposed to WNV vectors outside and in stables, and are spatially distributed near human settlements. Thus, equids can be sentinels for early detection of regional WNV activity (*4*).

In the Americas, WNV gained attention after its rapid spread in the United States beginning in 1999 (*4*). In South America, WNV dispersion is poorly understood. Seropositive horses were found in Colombia in 2004 (*5*) and in Argentina in 2006 (*6*). In Brazil, the largest country in South America, serologic studies from central, southeastern, and northeastern regions suggested WNV circulation among horses since at least 2009 (*7*,*8*). Human WNV infection was described only once, in 2014, from a patient in northeastern Brazil with encephalitis (*9*). In 2018, a WNV strain was isolated and sequenced during an epizootic in horses in the southeastern coast (*10*). The horse-derived virus from Brazil clustered with strains detected in different birds in the United States in 2002 and 2005 (*10*), indicating that migratory birds could play a role in WNV transmission in Brazil. 

Serologic WNV data from equids along avian migratory routes are scarce. In the only available study from northeastern Brazil, 1/88 horses was WNV seropositive with a low neutralization titer (*7*). In the absence of testing for cocirculating flaviviruses, a low WNV antibody titer could be caused infections with other flaviviruses eliciting cross-reactive antibodies (*11*). We conducted a seroepidemiologic study among equids to investigate the spread of WNV in northeastern Brazil. 

## The Study

**Figure 1:**
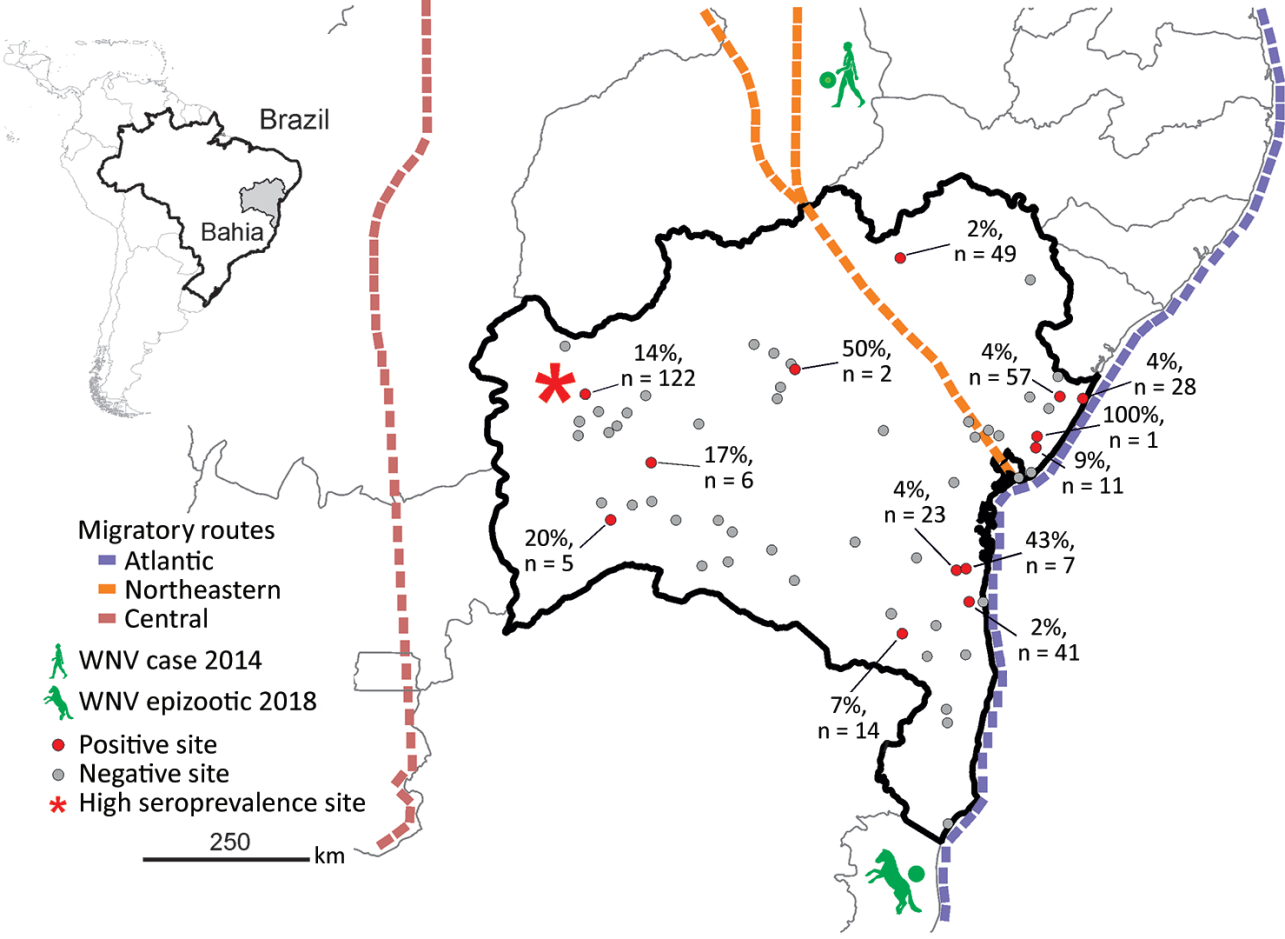
Geographic distribution and PRNT_90_–validated West Nile virus seroprevalence among equids per sampling site in Bahia State, Brazil. Inset shows location of Bahia State in northeastern Brazil. Sample sizes are shown only for locations with seropositive animals. Avian migratory routes are based on the 2016 annual report of the Chico Mendes Institute for Biodiversity and Conservation (https://www.researchgate.net/publication/292980285_Annual_Report_of_Flyways_and_Priority_Areas_for_Migratory_Birds_in_Brazil_Relatorio_anual_de_rotas_e_areas_de_concentracao_de_aves_migratorias_no_Brasil). PRNT_90_, plaque-reduction neutralization tests with a 90% endpoint.

We collected serum samples from 713 equids, including horses and mules, sampled as part of routine veterinary surveillance activities during 2013–2018 in the state of Bahia in northeastern Brazil. The animal ethics committee of the Federal University of Bahia approved the sampling and analyses (authorization no. 55/2017). Sampling covered a large area that connects the location of the human case from 2014 and the 2018 horse epizootic (*9*,*10*). The area is adjacent to the Atlantic, northeastern, and central avian migratory routes (Figure 1).

**Figure 2:**
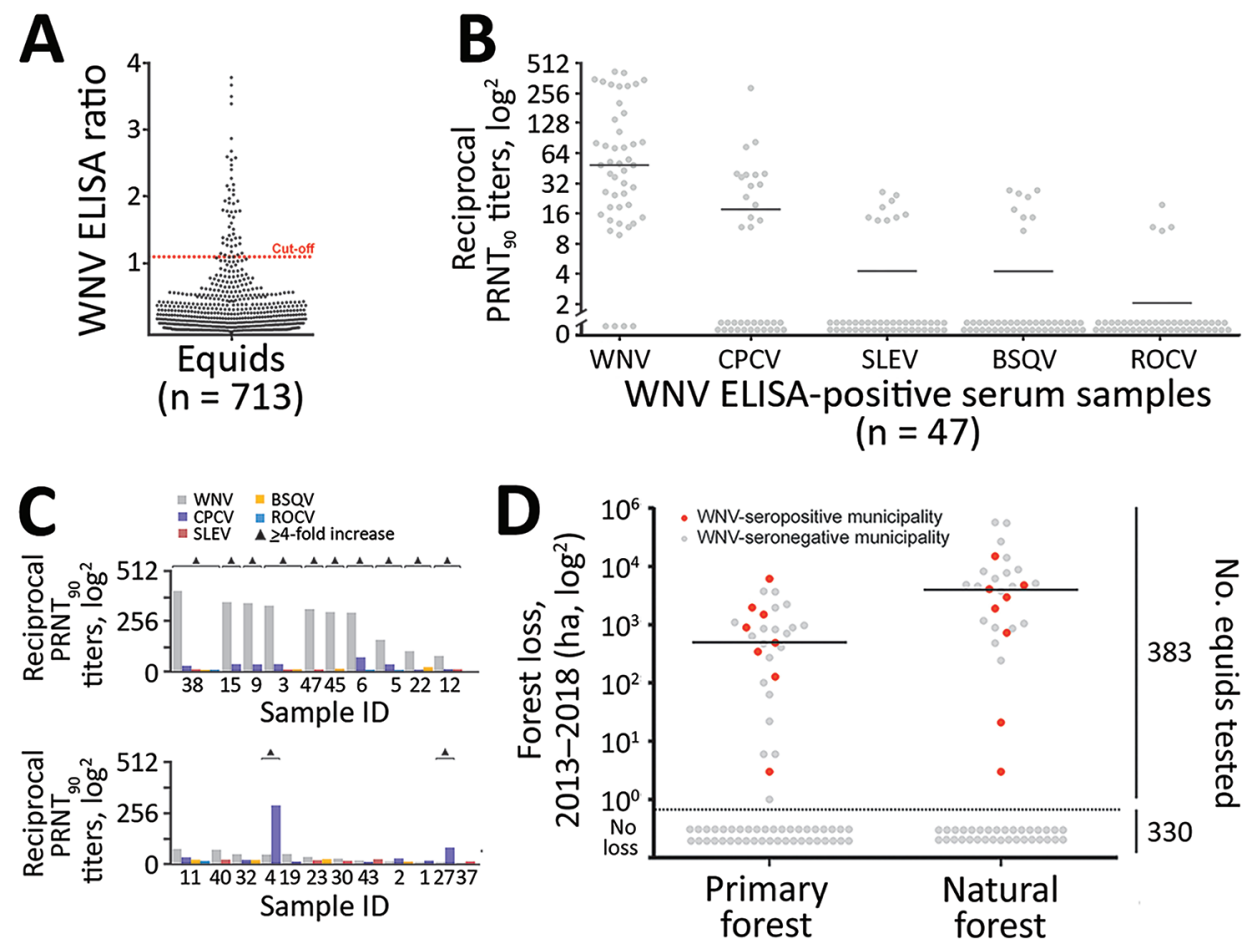
WNV seroprevalence among equids, Brazil. A) ELISA absorbance values displayed as sample to cutoff ratio, as previously described (*2*). We increased the ELISA cutoff by 10% above which samples were considered positive to maximize specificity because the ELISA was not originally validated for horses in Latin America, which are infected by more Japanese encephalitis serocomplex viruses compared with horses in Europe. Dotted orange line represents the 1.1 positivity cutoff. B) Reciprocal PRNT_90_ titers for WNV and other flaviviruses. Statistical significance levels were inferred by using the Kruskal-Wallis test. Bars indicate mean. Graph created by using Prism (GraphPad software, https://www.graphpad.com). C) Distinction of heterotypic serum samples based on the endpoint titers of various flaviviruses. Triangles indicated endpoint titers >4-fold. D) Effects of forests and forest loss on WNV seropositivity and seronegativity among equids in municipalities, Brazil. Natural forest is made up of introduced or native tree or vegetation that have reproduced naturally, without help or (human) intervention. Primary forest is made up of intact and nonintact natural forest and refers to areas that reached the final stage of succession during 2013–2018. Data on primary and natural forest were retrieved from Global Forest Watch (http://www.globalforestwatch.org). Right y-axis represents number of total number of equids tested for seroprevalence. Horizontal bars indicate means. Areas below dotted line had no forest loss. BSQV, Bussuquara virus; CPCV, Cacipacoré virus; ha, hectare (10,000 m^2^); PRNT_90_, 90% plaque-reduction neutralization test; ROCV, Rocio virus; SLEV, Saint Louis encephalitis virus; WNV, West Nile virus.

For antibody screening, we used an experimental WNV IgG ELISA based on a fusion loop envelope antigen containing mutations. We chose this ELISA to decrease the chances of cross-reactivity with antibodies elicited by other flaviviruses (*2*). Among 713 serum samples, 47 (6.6%, 95% CI 4.9%–8.7%) yielded positive ELISA results (Figure 2, panel A). Beyond WNV, horses in Latin America frequently are infected with Saint Louis encephalitis virus (SLEV), Cacipacoré virus (CPCV), Rocio virus (ROCV), and Bussuquara virus (BSQV) (*12*); and WNV, CPCV, and SLEV all belong to the Japanese encephalitis serocomplex (Appendix Figure 1). Serologic analyses for WNV-specific antibodies in horses could be compromised by cross-reactive antibodies against other flaviviruses, eliciting potentially false-positive test results (*11*). Therefore, we confirmed ELISA-based WNV antibody detection by comparing the endpoint titers for the 90% plaque-reduction neutralization tests (PRNT_90_), considered the standard for arbovirus serologic testing, for WNV, CPCV, SLEV, BSQV, and ROCV in all 47 ELISA-positive serum samples. Of the 47 samples, 20 (44.7%) neutralized WNV only in PRNT_90_; another 22 (46.8%) showed heterotypic reactions for WNV, CPCV, or SLEV (Figure 2, panel B). Averaged endpoint titers were significantly higher for WNV than for the other flaviviruses (p<0.0001) and exceeded those for CPCV, SLEV, BSQV, or ROCV by >4-fold in 12/22 heterotypic samples (Figure 2, panel C), a titer difference commonly considered decisive in flavivirus serology. Thus, 68.1% (32/47) of ELISA-positive samples had WNV-specific antibody responses (Figure 2, panel C); 4 samples were seronegative for all 5 flaviviruses by PRNT_90_, potentially because of differential sensitivity of ELISA and PRNT. No samples had higher SLEV-, BSQV-, or ROCV-specific PRNT_90_ titers compared with WNV, but 2 ELISA-positive samples had >4-fold endpoint titers for CPCV compared with WNV and other flaviviruses (Appendix Table 1). These findings substantiated WNV and CPCV cocirculation among equids in northeastern Brazil, which is consistent with previous data on CPCV circulation in another region of Brazil (*12*), and high specificity of the ELISA-based screening algorithm.

PRNT_90_ validated the overall WNV seroprevalence of 4.5% (32/713 samples; 95% CI 3.1%–6.3%), which we used for downstream analyses (Table 1). We noted seropositive animals in 11/28 municipalities distributed over ≈900 km^2^, suggesting wide geographic spread of WNV (Table 1; Figure 1). We observed a concentration of positive samples in 2018 (Appendix Figure 2), and in 1 municipality (Figure 1), comprising 9 different seropositive farms with an average seroprevalence of 13.9% (95% CI 8.3%–21.4%). Antibody levels typically decline over time after flavivirus infection (*13*), which might bias positivity rates of serologic tests. However, WNV-specific PRNT_90_ endpoint titers were significantly lower in 2018 than in the preceding years by Mann-Whitney test (p = 0.002), excluding a bias from hypothetically more recent WNV infection in the animals sampled in 2018 (Appendix Figure 3).

**Table 1:** West Nile virus seroprevalence per municipality, Brazil

Municipalities	Sampling year(s)	No.	% Seroprevalence (95% CI)*
Antonio Cardoso	2015, 2016	10	0
Barreiras	2014, 2017, 2018	17	0
Caatiba	2018	19	0
Conceição do Jacuípe	2013	29	0
Conde	2013	28	3.6 (0.1–18.4)
Cotegipe	2013	11	0
Cristopolis	2013	10	0
Esplanada	2013	57	3.5 (0.4–12.1)
Eunápolis	2013, 2014	21	0
Feira de santana	2013	29	0
Formosa do Rio Preto	2013, 2017	37	0
Gongogi	2018	23	4.3 (0.1–21.9)
Ibotirama	2013	6	0
Igaporã	2013	27	0
Itabela	2013	6	0
Itabuna	2013, 2017	41	2.4 (0.1–12.9)
Itaju do Colônia	2013, 2015	6	0
Itapetinga	2018	14	7.1 (0.2–33.9)
Jaborandi	2017	5	20.0 (0.5–71.6)
Juazeiro	2013, 2017	49	2.0 (0.5–14.0)
Lauro de Freitas	2017	14	0
Mata de São João	2015, 2016, 2017	11	9.1 (0.2–41.3)
Mucuri	2013	13	0
Palmas de Monte Alto	2013	18	0
Riachão das Neves	2017, 2018	122	13.9 (8.3–21.4)
Rio Real	2013	25	0
Serra Dourada	2017	6	16.7 (0.4–64.1)
Ubaitaba	2018	7	42.9 (9.9–81.6)
Others†	2013–2018	52	3.8 (0.5–13.2)
Total	2013–2018	713	4.5 (3.1–6.3)

*Seroprevalence is based on 90% endpoint plaque-reduction neutralization tests. †Detailed information, including municipalities with >5 serum samples, is available in Appendix Table 2).

We performed generalized linear model analyses and principal component analysis to compare 12 environmental, ecologic, and demographic factors potentially affecting WNV seroprevalence (Table 2; Appendix Figure 4). Anthropogenic changes of pristine habitats can increase the abundance of zoonotic pathogens (*14*), potentially including arboviruses like WNV (*15*). Indeed, the model considering forest loss, but not the model considering tree cover alone, was significantly associated with higher WNV seroprevalence (odds ratio [OR] 5.106, 95% CI 1.318–31.796; p = 0.005) (Table 2). Model results were consistent with a higher proportion of WNV-seropositive sites in disturbed areas compared with pristine areas by χ^2^ test (p = 0.009) (Figure 2, panel D). Higher WNV seroprevalence in disturbed areas was not biased by the number of animals living in those sites compared to sites from pristine areas. By Student *t*-test, neither the overall number of animals nor the number of animals per site differed significantly between disturbed (p = 0.9) and pristine areas (p = 0.2894) (Figure 2, panel D; Appendix Figure 5).

**Table 2:** Mathematical modeling of ecologic factors potentially affecting West Nile virus seroprevalence, Brazil*

Model	AIC	ΔAIC	AW	p value†	OR (95% CI)	Maximum OR difference among study sites‡	ρ§	Comment#
Distance to bird route, km								
Coastal	248.02	0.00	0.56	0.001	1.002 (1.001–1.004)	4.527	0.09	+
Northeastern	251.41	3.39	0.10	0.009	1.003 (1.001–1.006)	6.813	0.08	+
Central	252.17	4.16	0.07	0.014	0.999 (0.997–1.000)	4.545	−0.08	–
Forest loss, y/n	250.38	2.37	0.17	0.005	5.106 (1.518–31.796)	5.106	0.09	+
Presence of natural or primary forest, y/n	253.39	5.38	0.04	0.029	3.186 (1.111–13.48)	3.186	0.08	+
Altitude, m	255.53	7.51	0.01	0.105	1.002 (1.000–1.004)	3.518	0.06	+
Mean temperature, °C	258.03	10.01	0.00	0.719	0.876 (0.427–0.803)	1.613	−0.04	–
Hottest quarter	255.57	7.55	0.01	0.108	0.617 (0.347–1.113)	5.155	−0.04	–
Human density, no./km^2^	255.76	7.74	0.01	0.121	1.000 (1.000–1.001)	3.137	−0.01	+
Tree cover, %	256.87	8.86	0.01	0.257	0.981 (0.941–1.012)	2.618	−0.09	–
Horse density, no./km2	258.10	10.09	0.00	0.817	0.969 (0.741–1.275)	1.170	−0.03	–
Mean precipitation, mm	258.15	10.14	0.00	0.948	1.000 (0.999–1.001)	1.047	−0.01	–

*Models are sorted by AIC, an estimator of the model’s quality; models with lower AIC values are superior to models with higher AIC values. Horse and human densities were based on 2018 data available from the Brazilian Institute of Geography and Statistics (https://www.ibge.org.br). Information on precipitation and mean temperature was obtained from WorldClim version 2 (https://www.worldclim.org). Information on tree cover was obtained from Copernicus Global Land Cover (https://lcviewer.vito.be/download). Information on natural or primary forest loss was obtained from Global Forest Watch (https://www.globalforestwatch.org). AIC, Akaike information criterion; AW, Akaike weight; OR, odds ratio; ΔAIC, the difference between a given and the best-supported model in AIC. †p values were determined by likelihood ratio tests of the different models. ‡Maximum OR difference among study sites indicates the highest OR difference possible for a given variable for better comparability between binary and nonbinary variables. §ρ, the Spearman correlation coefficient, ranges between −1 for negative correlations and 1 for positive correlations. The closer ρ is to 1 or −1, the greater the correlation between the observed variables. #Clarification that the observed variable is associated with an increase (+) or a decrease (–) of West Nile virus prevalence.

Because the geographic distribution of both the 2018 horse epizootic and the only known human case might be linked geographically to the northeastern and coastal avian migratory routes (Figure 1), we included distances to avian routes in model analyses of WNV seroprevalence. Proximity to the central avian migratory route was associated with higher WNV seroprevalence (Table 2; Appendix Figure 4). This finding was consistent with other seroprevalence studies, indicating the presence of WNV in horses in the central region in Brazil (*7*,*8*), but failed to connect the WNV detections in Brazil to geographically adjacent avian migratory routes. Our data were consistent with prior studies of WNV ecology, but the explicatory power of our models was low despite statistical significance (Table 2; Appendix Figure 4).

Our study was limited by absence of longitudinal samples from individual sampling sites, lack of information on animal trade and animal age, and relatively low numbers of seropositive animals from individual sites. Thus, we cannot exclude biases affecting the accuracy of our modeling approach. However, our large sample and the combination of thorough serologic analyses and mathematical modeling enabled robust estimates of WNV spread that can guide prospective studies.

## Conclusions

Our findings of substantial WNV seroprevalence in equids in Brazil warrants WNV surveillance in cases of acute neurologic disease in humans and horses. In addition, blood products should be screened in areas of Latin America with high risk for WNV.

Appendix Additional information ecologic determinants of West Nile virus seroprevalence among equids, Brazil.
