# Supplementary material for: Ecologic Determinants of West Nile Virus Seroprevalence among Equids, Brazil
Source: Emerg Infect Dis. 2021 Sep;27(9):2466–70. doi: 10.3201/eid2709.204706 (PMC8386811; doi:10.3201/eid2709.204706)
Supplement: Appendix — Additional information ecologic determinants of West Nile virus seroprevalence among equids, Brazil. [file 20-4706-Techapp-s1.pdf]

Article DOI: https://doi.org/10.3201/eid2709.204706

Ecologic Determinants of West Nile Virus Seroprevalence among Equids, Brazil

Appendix

Appendix Methods

Samples

During routine surveillance activities conducted by local animal health agencies during 2013–2018, a total of 713 serum samples were collected from 618 horses and 95 mules. Animals were sampled in a large geographic area comprising 61 of the 417 municipalities in the state of Bahia.

Serologic Detection

After initial ELISA-based screening, 47 positive serum samples were tested by using plaque-reduction neutralization tests with a 90% endpoint ($PRNT_{90}$). Samples were tested for specific neutralizing antibodies against West Nile virus (WNV) by using strain NY-99, Cacipacoré virus (CPCV) by using strain UVE/CPCV/1977/BR/Be An 327600, Saint Louis encephalitis virus (SLEV) by using strain MSI-7, Bussuquara virus (BSQV) by using strain BeAn 4073, and Rocio virus (ROCV) by using strain UVE/ROCV/1975/BR/5P H34 675. $PRNT_{90}$ was conducted in monolayers of 1.6×10^5 VeroFM cells for WNV and BSQV, 1.6×10^5 VeroB4 cells for ROCV, 3.5×10^5 HD11 (CCLV-RIE 1510) cells derived from chicken macrophages for CPCV, or 1.2×10^5 baby hamster kidney 21 (BHK-21) cells for SLEV, seeded in 12-well plates 1 day before the infection. We incubated 50 plaque forming units (PFUs) with serum dilutions for 1 h and added to the cell monolayer. After the 1-hour incubation, we added an overlayer containing Dulbecco's Modified Eagle Medium (DMEM; Gibco, https://www.thermofisher.com) with 2% fetal calf serum (FCS; Gibco), and 2.5% carboxymethyl cellulose (CMC; Carl Roth, https://www.carlroth.com). The medium was removed after 2 days for WNV, after 4 days for SLEV, after 5 days for CPCV and BSQV, or after 6 days for ROCV; cells were fixed with 6% paraformaldehyde and stained with crystal violet. The endpoint titration

of positive serum samples was done by testing 1:40, 1:80, 1:160, 1:320, 1:640, 1:1,280, 1:2,560 and 1:5,120 serum dilutions; we considered titers reducing the number of PFU by ≥90% positive. Titers were calculated using the logistic regression function in Prism 6 (GraphPad software, https://www.graphpad.com).

Principal Component Analysis

We conducted a principal component analysis (PCA) by using the complete sample dataset to visualize the structure of the dataset and the relationship of different variables. For the PCA, we removed any unneeded variables from the dataset. We used R version 4.0.2 (R Foundation for Statistical Computing, https://www.r-project.org) and factoextra version 1.0.7 (BEAR Applications, University of Birmingham, UK, https://bear-apps.bham.ac.uk) to conduct PCA. We used cosign-squared (cos^2) values to color arrows for better visualization.

Generalized Linear Model Analyses

We calculated generalized linear models (GLMs) GLMs to compare the effects of environmental, ecologic, and demographic factors on the seroprevalence of WNV. After initial testing, we selected 12 variables for further analyses on the basis of Akaike information criterion (AIC) values and their reasonability; for example, we selected only the precipitation variable having the best AIC support. We calculated GLMs, the statistical support of the GLMs, and odds ratios (95% CI) in R version 4.0.2 (R Foundation for Statistical Computing) by using the glm() function and we conducted a Spearman correlation test by using the cor.test() function. We analyzed AIC values by using the bbmle version 1.0.23.1 package in R (R Foundation for Statistical Computing).

Appendix Table 1. Comparative plaque-reduction neutralization test results for West Nile virus ELISA-positive serum samples from equids, Brazil*

Sample ID	Species	Municipality	ELISA OD	ELISA ratio	$PRNT_{90}$ endpoint titers: WNV	CPCV	SLEV	BSQV	ROCV	$PRNT_{90}$ interpretation	Sampling year
ZS-A-1367	Horse	Barreiras	0.521	1.4	1:14	1:20	<10	<10	<10	Undetermined	2018
ZS-A-1246	Horse	Caatiba	0.547	1.4	1:16	1:31	<10	1:15	<10	Undetermined	2018
ZS-A-19	Horse	Conde	0.953	2.5	1:343	1:41	1:14	1:15	<10	WNV	2013
ZS-A-224	Horse	Esplanada	1.001	2.6	1:50	1:296	<10	<10	<10	CPCV	2013
ZS-A-237	Horse	Esplanada	0.652	1.7	1:166	1:40	<10	<10	1:11	WNV	2013
ZS-A-239	Horse	Esplanada	0.582	1.5	1:307	1:76	<10	<10	1:12	WNV	2013
ZS-A-1201	Mule	Gongogi	0.525	1.4	1:33	<10	<10	<10	<10	WNV	2018
ZS-A-789	Horse	Itabuna	0.682	1.8	1:26	<10	<10	<10	<10	WNV	2013
ZS-A-1219	Horse	Itapetinga	0.995	2.6	1:356	1:40	<10	<10	<10	WNV	2018
ZS-A-1221	Horse	Itapetinga	0.453	1.2	<10	<10	<10	<10	<10	Negative	2018
ZS-A-819	Horse	Jaborandi	0.685	1.8	1:81	1:38	<10	1:26	1:20	Undetermined	2017
ZS-A-820	Horse	Jaborandi	0.534	1.4	1:83	1:15	1:15	<10	<10	WNV	2017
ZS-A-1108	Mule	Juazeiro	0.531	1.4	1:85	<10	<10	<10	<10	WNV	2013
ZS-A-481	Horse	Lapão	0.687	1.8	1:209	<10	<10	<10	<10	WNV	2014
ZS-A-125	Horse	Mata de São João	0.618	1.6	1:361	1:41	<10	<10	<10	WNV	2015
ZS-A-127	Horse	Pojuca	0.734	1.9	1:143	<10	<10	<10	<10	WNV	2016

Sample ID	Species	Municipality	ELISA OD	ELISA ratio	PRNT$_{90}$ endpoint titers					PRNT$_{90}$ interpretation	Sampling year
					WNV	CPCV	SLEV	BSQV	ROCV		
ZS-A-1257	Horse	Riachão das Neves	1.458	3.8	1:57	<10	<10	<10	<10	WNV	2018
ZS-A-1258	Horse	Riachão das Neves	1.306	3.4	1:10	<10	<10	<10	<10	WNV	2018
ZS-A-1259	Horse	Riachão das Neves	1.106	2.9	1:50	1:12	<10	<10	<10	WNV	2018
ZS-A-1263	Horse	Riachão das Neves	1.033	2.7	1:27	<10	<10	<10	<10	WNV	2018
ZS-A-1266	Horse	Riachão das Neves	0.983	2.6	1:78	<10	<10	<10	<10	WNV	2018
ZS-A-1267	Horse	Riachão das Neves	0.927	2.4	1:108	1:12	<10	1:26	<10	WNV	2018
ZS-A-1268	Horse	Riachão das Neves	0.876	2.3	1:38	<10	1:22	1:28	<10	Undetermined	2018
ZS-A-1272	Horse	Riachão das Neves	0.84	2.2	1:11	<10	<10	<10	<10	WNV	2018
ZS-A-1276	Horse	Riachão das Neves	0.833	2.2	<10	<10	<10	<10	<10	Negative	2018
ZS-A-1281	Horse	Riachão das Neves	0.81	2.1	1:44	<10	<10	<10	<10	WNV	2018
ZS-A-1283	Horse	Riachão das Neves	0.808	2.1	1:13	1:85	<10	<10	<10	CPCV	2018
ZS-A-1299	Horse	Riachão das Neves	0.782	2.0	1:54	<10	<10	<10	<10	WNV	2018
ZS-A-1303	Horse	Riachão das Neves	0.76	2.0	1:19	<10	<10	<10	<10	WNV	2018
ZS-A-1304	Mule	Riachão das Neves	0.746	1.9	1:30	<10	1:19	<10	<10	Undetermined	2018
ZS-A-1307	Horse	Riachão das Neves	0.745	1.9	1:75	<10	<10	<10	<10	WNV	2018
ZS-A-1311	Horse	Riachão das Neves	0.731	1.9	1:52	1:24	<10	1:24	<10	Undetermined	2018
ZS-A-1316	Horse	Riachão das Neves	0.693	1.8	1:41	<10	<10	<10	<10	WNV	2018
ZS-A-1321	Horse	Riachão das Neves	0.688	1.8	1:19	<10	<10	<10	<10	WNV	2018
ZS-A-1322	Horse	Riachão das Neves	0.596	1.5	1:25	<10	<10	<10	<10	WNV	2018
ZS-A-1327	Horse	Riachão das Neves	0.555	1.4	1:432	<10	<10	<10	<10	WNV	2018
ZS-A-1328	Horse	Riachão das Neves	0.503	1.3	1:12	<10	1:16	<10	<10	Undetermined	2018
ZS-A-1329	Horse	Riachão das Neves	0.499	1.3	1:419	1:32	1:15	1:11	1:12	WNV	2018
ZS-A-1350	Horse	Riachão das Neves	0.49	1.3	<10	<10	<10	<10	<10	Negative	2018
ZS-A-1373	Mule	Riachão das Neves	0.489	1.3	1:74	<10	1:25	<10	<10	Undetermined	2018
ZS-A-1374	Mule	Riachão das Neves	0.484	1.3	<10	<10	<10	<10	<10	Negative	2018
ZS-A-1376	Mule	Riachão das Neves	0.443	1.1	1:13	<10	<10	<10	<10	WNV	2018
ZS-A-1378	Mule	Riachão das Neves	0.434	1.1	1:20	1:14	1:27	<10	<10	Undetermined	2018
ZS-A-842	Horse	Serra Dourada	0.66	1.7	1:329	<10	<10	<10	<10	WNV	2017
ZS-A-1186	Mule	Ubaitaba	1.414	3.7	1:310	<10	<10	1:18	<10	WNV	2018
ZS-A-1191	Mule	Ubaitaba	1.348	3.5	1:15	<10	<10	<10	<10	WNV	2018
ZS-A-1380	Horse	Ubaitaba	0.534	1.4	1:325	<10	1:14	<10	<10	WNV	2018

*BSQV, Bussuquara virus; CPCV, Cacipacoré virus; ID, identification; OD, optical density; PRNT$_{90}$, plaque-reduction neutralization test using a 90% endpoint; ROCV, Rocio virus; SLEV, Saint Louis encephalitis virus; WNV, West Nile virus.

Appendix Table 2. West Nile virus seroprevalence among equids per municipality, Brazil

Municipalities	Sampling year	No.	% Seroprevalence (95% CI)*
Angical	2013	1	0
Anguera	2017	1	0
Antonio Cardoso	2015–2016	10	0
Baianópolis	2018	1	0
Barra do Mendes	2014	1	0
Barreiras	2014, 2017, 2018	17	0
Caatiba	2018	19	0
Caetité	2014	1	0
Central	2014	1	0
Conceição do Jacuípe	2013	29	0
Conde	2013	28	3.6 (0.1–18.4)
Correntina	2017	1	0
Cotegipe	2013	11	0
Cristopolis	2013	10	0
Entre Rios	2017	1	0
Esplanada	2013	57	3.5 (0.4–12.1)
Eunápolis	2013, 2014	21	0
Feira de Santana	2013	29	0
Formosa do Rio Preto	2013, 2017	37	0
Gongogi	2018	23	4.3 (0.1–21.9)
Guanambi	2013	3	0
Ibipeba	2014	2	0
Ibotirama	2013	6	0
Igaporã	2013	27	0
Ilheus	2016, 2017	4	0
Inhambupe	2013	1	0
Irece	2014	2	0
Itabela	2013	6	0
Itabuna	2013, 2017	41	2.4 (0.1–12.9)
Itagi	2013	1	0
Itaguaçú da bahia	2017	1	0
Itaju do Colônia	2013, 2015	6	0
Itapetinga	2018	14	7.1 (0.2–33.9)
Jaborandi	2017	5	20.0 (0.5–71.6)
Jandaíra	2013	1	0
Jeremoabo	2016	1	0
Juazeiro	2013, 2017	49	2.0 (0.5–14.0)
Lagoa real	2013	2	0
Lapão	2014	2	50.0 (1.3–98.7)
Lauro de Freitas	2017	14	0
Malhada	2013	3	0
Mascote	2015	4	0
Mata de São João	2015, 2016, 2017	11	9.1 (0.2–41.3)
Mucuri	2013	13	0
Palmas de Monte Alto	2013	18	0
Pojuca	2016	1	100.0 (2.5–100.0)
Potiraguá	2013	1	0
Riachão das Neves	2017, 2018	122	13.9 (8.3–21.4)
Riacho de Santana	2014	1	0
Rio Real	2013	25	0
Ruy Barbosa	2013	2	0
Salvador	2013	1	0
Santa Maria da Vitória	2013	1	0
Santana	2017	1	0
São Desidério	2013	1	0
São Félix do Coribe	2017	2	0
São Miguel das Matas	2014	1	0
Serra do Ramalho	2014	1	0
Serra Dourada	2017	6	16.7 (0.4–64.1)
Ubaitaba	2018	7	42.9 (9.9–81.6)
Wanderley	2014	1	0
Total	2013–2018	713	4.5 (3.1–6.3)

*Based on plaque-reduction neutralization test using a 90% endpoint.

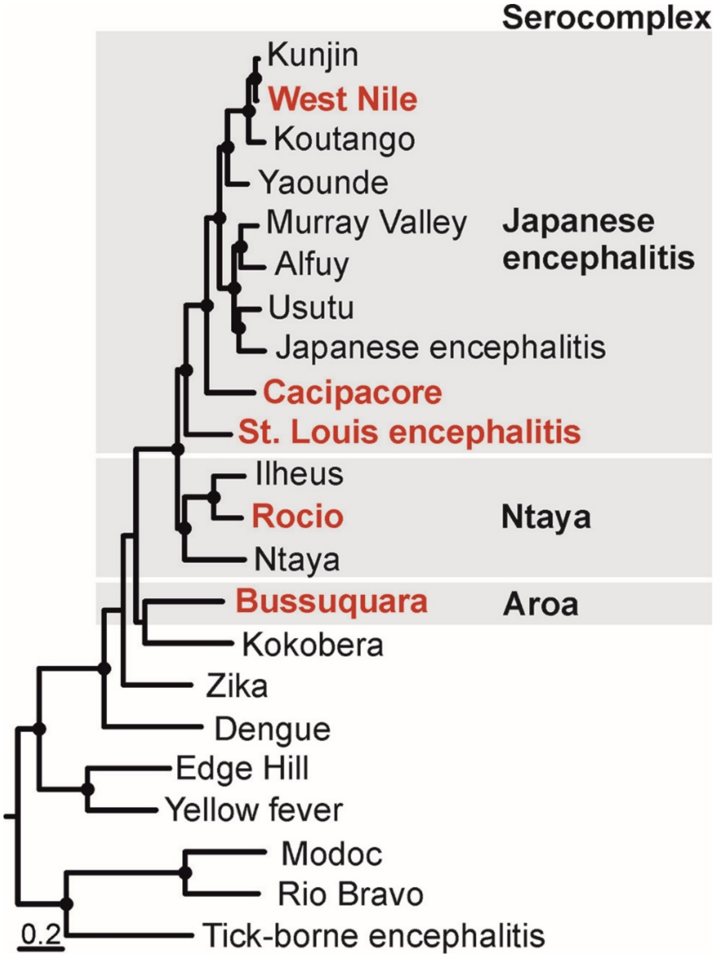

Appendix Figure 1. Maximum likelihood phylogenetic tree showing relevant flaviviruses used to assess West Nile virus seroprevalence among equids, Brazil. The tree was generated by using MEGA X (https://www.megasoftware.net) and a dataset comprising translated polyprotein genes and a Whelan and Goldman amino acid substitution model. Red text indicates flaviviruses from Brazil. Black dots indicate support values >0.70 from 500 bootstrap replicates. Scale bar indicates nucleotide substitutions per site.

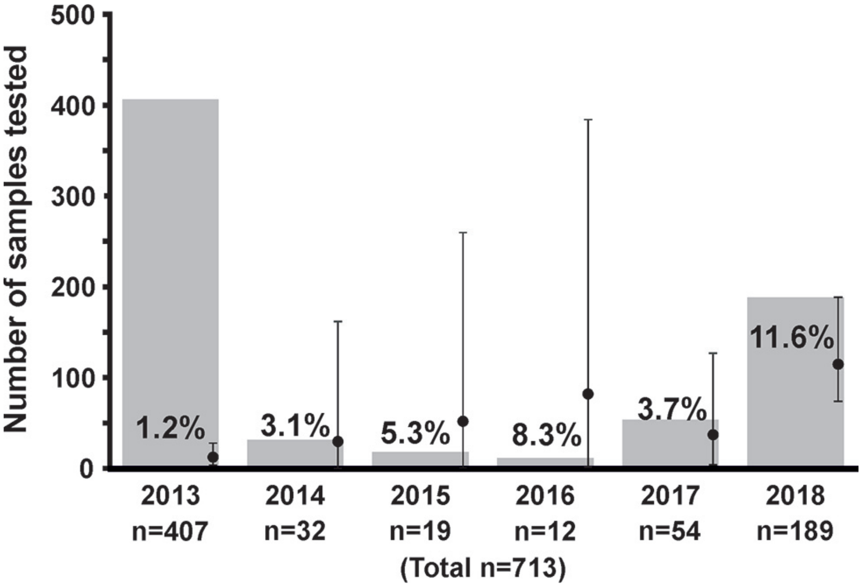

Appendix Figure 2. Seroprevalence of West Nile virus among equids over time, Brazil. Seroprevalence is based on plaque-reduction neutralization tests using a 90% endpoint ($PRNT_{90}$). Black dots indicate seroprevalence values of each year; vertical bars indicate 95% CI.

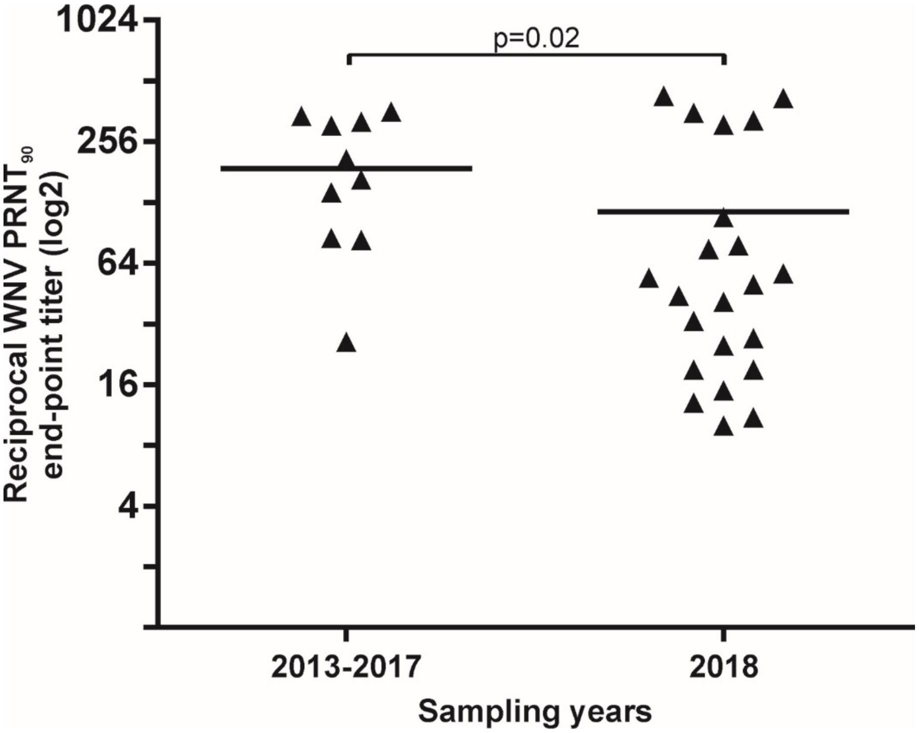

Appendix Figure 3. Comparison between 2018 and 2013–2017 West Nile virus–specific titers of plaque-reduction neutralization tests using a 90% endpoint ($PRNT_{90}$). Triangles represent $PRNT_{90}$–positive sera.

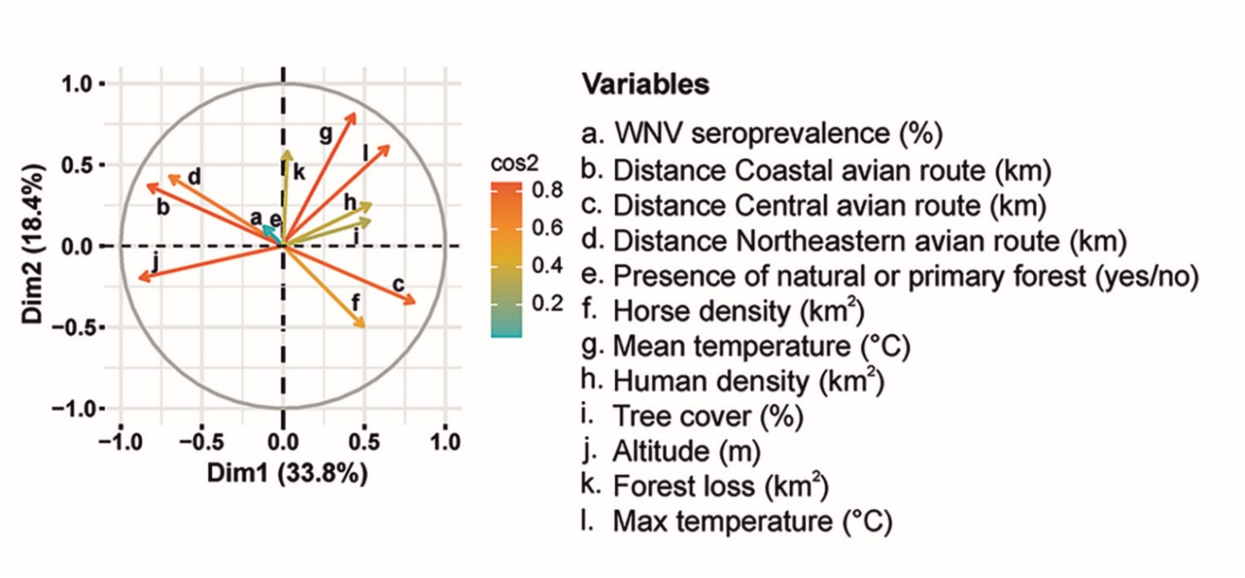

Appendix Figure 4. Principal component analysis in 2 dimensions (Dim) showing the relationship of variables used to model ecologic factors potentially affecting West Nile virus (WNV) seroprevalence, Brazil. Squared cosine (cos2) measures the interaction between 2 variables and is expressed in a gradient computing the effect of a principal component's contribution over a given observation.

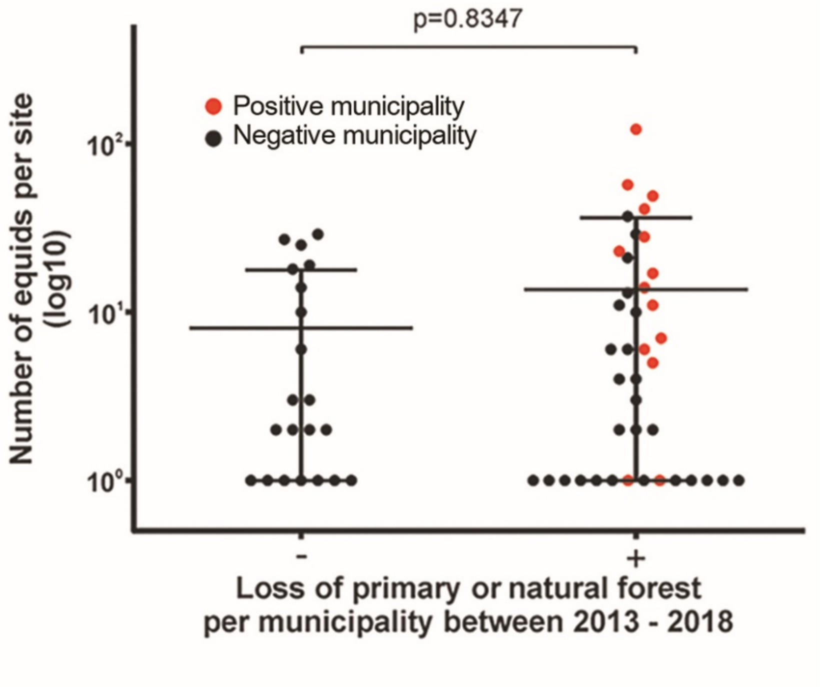

Appendix Figure 5. Number of equids per municipality with (+) and without (–) primary or natural forest loss. Bars indicate mean and standard deviation. Statistical significance was determined by Student *t*-test.
